# An ortho­rhom­bic polymorph of 3,4-di­amino­benzo­nitrile

**DOI:** 10.1107/S1600536813008489

**Published:** 2013-04-05

**Authors:** David K. Geiger, Dylan E. Parsons

**Affiliations:** a Department of Chemistry, State University of New York-College at Geneseo, 1 College Circle, Geneseo, NY 14454, USA

## Abstract

The title compound, C_7_H_7_N_3_, is an ortho­rhom­bic polymorph that crystallizes in the space group *Pca*2_1_. The previously reported monoclinic form [Geiger & Parsons (2013 ▶) *Acta Cryst.* E**69**, o452] crystallizes in the space group *P*2_1_/*c* (*Z* = 4). In the crystal, two independent HN—H⋯N C hydrogen bonds link the mol­ecules into chains along the *a*-glide plane. Two further independent HN—H⋯NH_2_ hydrogen bonds join the chains, forming a three-dimensional network.

## Related literature

For the structure of the monoclinic polymorph of the title compound, see: Geiger & Parsons (2013 ▶). For the structures of the two crystalline forms of 1,2-di­amino­benzene, see: Czapik & Gdaniec (2010 ▶); Stålhandske (1981 ▶).
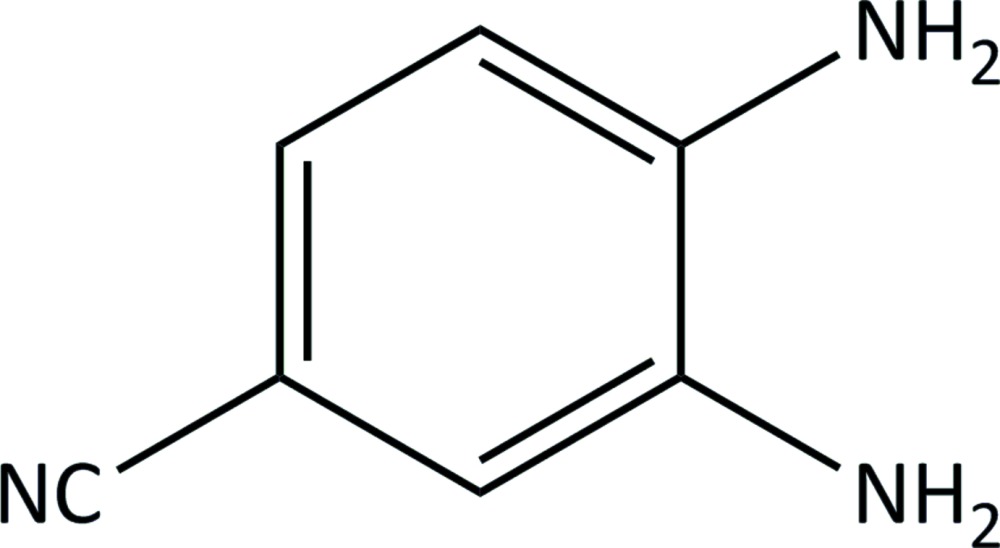


## Experimental

### 

#### Crystal data

C_7_H_7_N_3_
*M* *_r_* = 133.16
Orthorhombic, 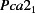

*a* = 17.425 (3) Å
*b* = 4.5225 (8) Å
*c* = 8.6167 (16) Å
*V* = 679.0 (2) Å^3^
*Z* = 4
Mo *K*α radiation
μ = 0.09 mm^−1^
*T* = 200 K
0.60 × 0.30 × 0.30 mm

#### Data collection

Bruker SMART X2S CCD diffractometer
Absorption correction: multi-scan (*SADABS*; Bruker, 2010 ▶) *T*_min_ = 0.84, *T*_max_ = 0.98
3295 measured reflections
652 independent reflections
598 reflections with *I* > 2σ(*I*)
*R*_int_ = 0.030

#### Refinement

*R*[*F* ^2^ > 2σ(*F* ^2^)] = 0.030
*wR*(*F* ^2^) = 0.081
*S* = 1.08
652 reflections
103 parameters
1 restraint
H atoms treated by a mixture of independent and constrained refinement
Δρ_max_ = 0.09 e Å^−3^
Δρ_min_ = −0.14 e Å^−3^

### 

Data collection: *APEX2* (Bruker, 2010 ▶); cell refinement: *SAINT* (Bruker, 2010 ▶); data reduction: *SAINT*; program(s) used to solve structure: *SHELXS97* (Sheldrick, 2008 ▶); program(s) used to refine structure: *SHELXL97* (Sheldrick, 2008 ▶); molecular graphics: *XSHELL* (Bruker, 2010 ▶) and *Mercury* (Macrae *et al.*, 2008 ▶); software used to prepare material for publication: *publCIF* (Westrip, 2010 ▶).

## Supplementary Material

Crystal structure: contains datablock(s) I, global. DOI: 10.1107/S1600536813008489/qk2056sup1.cif

Structure factors: contains datablock(s) I. DOI: 10.1107/S1600536813008489/qk2056Isup2.hkl

Supplementary material file. DOI: 10.1107/S1600536813008489/qk2056Isup3.mol

Supplementary material file. DOI: 10.1107/S1600536813008489/qk2056Isup4.cml

Additional supplementary materials:  crystallographic information; 3D view; checkCIF report

## Figures and Tables

**Table 1:** Hydrogen-bond geometry (Å, °)

*D*—H⋯*A*	*D*—H	H⋯*A*	*D*⋯*A*	*D*—H⋯*A*
N1—H1*A*⋯N3^i^	0.91 (3)	2.49 (3)	3.218 (3)	138 (2)
N1—H1*B*⋯N2^ii^	0.92 (3)	2.20 (3)	3.107 (3)	170 (3)
N2—H2*A*⋯N3^i^	0.95 (3)	2.22 (3)	3.152 (3)	168 (3)
N2—H2*B*⋯N1^iii^	0.89 (3)	2.41 (3)	3.210 (3)	149 (2)

Symmetry codes: (i) 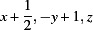; (ii) 
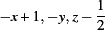
; (iii) 
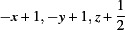
.

**Fig. 1.**
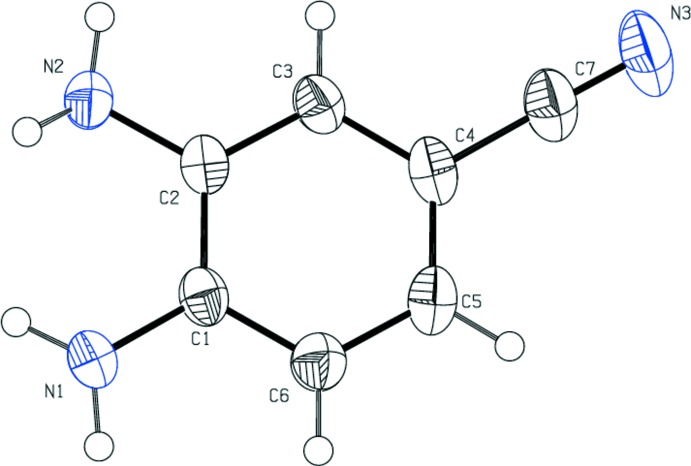
A view of the title compound showing the atom-labeling scheme. Thermal parameters are drawn at the 50% probability level.

**Fig. 2.**
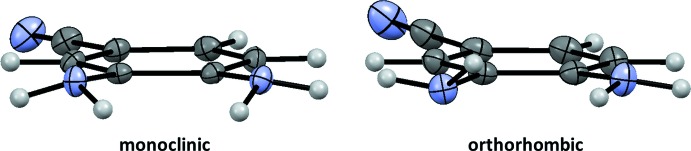
A view of the amine stereochemistry exhibited by the two polymorphs of the title compound.

**Fig. 3.**
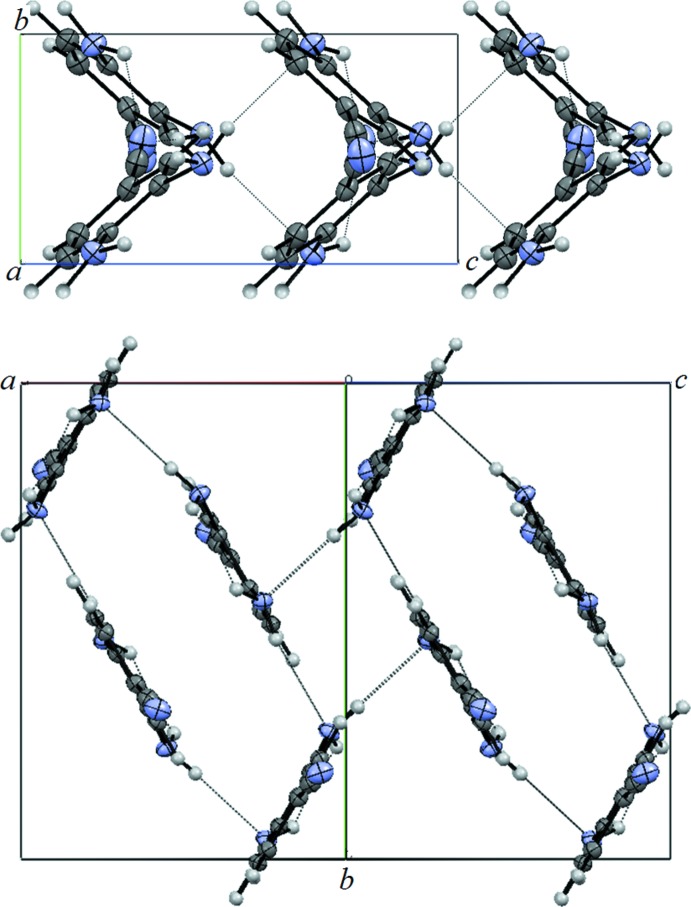
A view down [100] of the orthorhombic polymorph (top) and down [101] of the monoclinic polymorph showing the H-bonding network.

## supplementary crystallographic information

### Comment

Single crystals of the title compound were obtained by slow evaporation of an ethanolic solution of commercially available 3,4-diaminobenzonitrile. The monoclinic (Geiger & Parsons, 2013) and the orthorhombic polymorphs were obtained from the same batch of crystals. Interestingly, monoclinic (Stålhandske, 1981) and orthorhombic (Czapik & Gdaniec, 2010) polymorphs of the unsubstituted 1,2-diaminobenzene have also been reported.

Figure 1 shows a perspective view of the title compound with the atom numbering scheme. The non-hydrogen atoms of the molecule are essentially planar with a r.m.s. deviation of 0.0449 Å and a maximum deviation of 0.0915 (13) Å for N1. As shown in Fig. 2, in the monoclinic polymorph (Geiger & Parsons, 2013), the amine groups are oriented on the same side of the benzene ring. In contrast, in the orthorhombic form (Fig. 2), they are directed toward opposite sides of the benzene plane. N1 and N2 are 0.072 (2) and 0.074 (2) Å, respectively, out of the plane. In the monoclinic form, the amine groups are decidedly pyramidal whereas the H—N—H angles are 120 (2)° and 116 (2)° for N1 and N2, respectively, in the orthorhomic polymorph. H1A and H1B are 0.10 (2) and 0.09 (2) Å out of the benzene plane and H2A and H2B are 0.42 (2) and 0.08 (2) Å out of the plane. The nitrile group is linear with C3—C7—N3 = 178.5 (2)°.

A comparison of the H-bonding network in the two polymorphs is shown in Fig. 3. Both polymorphs exhibit N—H···N≡C hydrogen bonding involving both of the amines, which results in chains of molecules (along the *a*-glide plane for the orthorhomic form and along [1 0 1] in the monoclinic form). However, in the monoclinic polymorph, the molecules in the chain are coplanar while in the orthorhombic polymorph subsequent molecules in the chain subtend an angle of 82.0°. The chains are linked by a network of HN—H···NH_2_ hydrogen bonds in both forms. In contrast, in the two known polymorphs of 1,2-diaminobenzene (Stålhandske, 1981; Czapik & Gdaniec, 2010), one of the N—H bonds of each amine group is coplanar with the benzene ring and an intramolecular N—H···N interaction is exhibited. Intermolecular hydrogen bonding results in layers that are joined by additional hydrogen-bonding interactions. No intramolecular hydrogen bonding is observed in either polymorph of the title compound.

### Experimental

Single crystals of the title compound were obtained by slow evaporation of an ethanolic solution of commercially available 3,4-diaminobenzonitrile.

### Refinement

The H atoms bonded to carbon were refined using a riding model with C—H = 0.95 Å and *U*_iso_ = 1.2*U*_eq_(C). The coordinates and isotropic thermal parameters of the amine H atoms were refined freely. A negative (meaningless) Flack parameter and corresponding standard deviation were observed. Inversion of the structure also gave nonsensical results for the Flack parameter and use of TWIN and BASF resulted in a negative *x* parameter. In the absence of significant anomalous scattering, Friedel pairs were merged (MERG 3) and the absolute structure was assigned arbitrarily. Although merging the Friedel pairs gives a poorer data/parameter ratio, the higher ratio with retention of Friedel opposites is illusory.

### Crystal data

**Table:**

C_7_H_7_N_3_	*D*_x_ = 1.302 Mg m^−^^3^
*M**_r_* = 133.16	Mo *K*α radiation, λ = 0.71073 Å
Orthorhombic, *P**c**a*2_1_	Cell parameters from 1493 reflections
*a* = 17.425 (3) Å	θ = 3.3–24.9°
*b* = 4.5225 (8) Å	µ = 0.09 mm^−^^1^
*c* = 8.6167 (16) Å	*T* = 200 K
*V* = 679.0 (2) Å^3^	Pyramidal, colourless
*Z* = 4	0.60 × 0.30 × 0.30 mm
*F*(000) = 280	

### Data collection

**Table:**

Bruker SMART X2S CCD diffractometer	652 independent reflections
Radiation source: fine-focus sealed tube	598 reflections with *I* > 2σ(*I*)
Graphite monochromator	*R*_int_ = 0.030
Detector resolution: 8.33 pixels mm^-1^	θ_max_ = 25.1°, θ_min_ = 3.3°
ω scans	*h* = −20→12
Absorption correction: multi-scan (*SADABS*; Bruker, 2010)	*k* = −5→5
*T*_min_ = 0.84, *T*_max_ = 0.98	*l* = −10→10
3295 measured reflections	

### Refinement

**Table:**

Refinement on *F*^2^	Primary atom site location: structure-invariant direct methods
Least-squares matrix: full	Secondary atom site location: difference Fourier map
*R*[*F*^2^ > 2σ(*F*^2^)] = 0.030	Hydrogen site location: inferred from neighbouring sites
*wR*(*F*^2^) = 0.081	H atoms treated by a mixture of independent and constrained refinement
*S* = 1.08	*w* = 1/[σ^2^(*F*_o_^2^) + (0.0563*P*)^2^ + 0.0095*P*] where *P* = (*F*_o_^2^ + 2*F*_c_^2^)/3
652 reflections	(Δ/σ)_max_ < 0.001
103 parameters	Δρ_max_ = 0.09 e Å^−^^3^
1 restraint	Δρ_min_ = −0.14 e Å^−^^3^

### Special details

**Table:**

Geometry. All e.s.d.'s (except the e.s.d. in the dihedral angle between two l.s. planes) are estimated using the full covariance matrix. The cell e.s.d.'s are taken into account individually in the estimation of e.s.d.'s in distances, angles and torsion angles; correlations between e.s.d.'s in cell parameters are only used when they are defined by crystal symmetry. An approximate (isotropic) treatment of cell e.s.d.'s is used for estimating e.s.d.'s involving l.s. planes.
Refinement. Refinement of *F*^2^ against ALL reflections. The weighted *R*-factor *wR* and goodness of fit *S* are based on *F*^2^, conventional *R*-factors *R* are based on *F*, with *F* set to zero for negative *F*^2^. The threshold expression of *F*^2^ > σ(*F*^2^) is used only for calculating *R*-factors(gt) *etc*. and is not relevant to the choice of reflections for refinement. *R*-factors based on *F*^2^ are statistically about twice as large as those based on *F*, and *R*- factors based on ALL data will be even larger.

### Fractional atomic coordinates and isotropic or equivalent isotropic displacement parameters (Å^2^)

**Table:**

	*x*	*y*	*z*	*U*_iso_*/*U*_eq_	
N1	0.50605 (11)	0.0476 (4)	0.6680 (3)	0.0412 (5)	
H1A	0.5442 (16)	0.085 (5)	0.737 (4)	0.062*	
H1B	0.5095 (15)	−0.112 (6)	0.602 (4)	0.062*	
N2	0.47588 (10)	0.4365 (4)	0.9148 (2)	0.0373 (4)	
H2A	0.5230 (17)	0.459 (5)	0.861 (4)	0.056*	
H2B	0.4617 (15)	0.591 (6)	0.972 (4)	0.056*	
N3	0.14367 (11)	0.5278 (5)	0.7794 (3)	0.0640 (7)	
C1	0.43184 (9)	0.1346 (4)	0.6990 (2)	0.0327 (5)	
C2	0.41581 (10)	0.3421 (4)	0.8177 (2)	0.0317 (5)	
C3	0.34103 (10)	0.4346 (4)	0.8399 (3)	0.0354 (5)	
H3	0.3300	0.5737	0.9194	0.043*	
C4	0.28135 (10)	0.3268 (4)	0.7471 (2)	0.0381 (5)	
C5	0.29684 (10)	0.1223 (5)	0.6311 (3)	0.0409 (5)	
H5	0.2565	0.0481	0.5680	0.049*	
C6	0.37141 (12)	0.0272 (4)	0.6082 (3)	0.0397 (5)	
H6	0.3818	−0.1138	0.5292	0.048*	
C7	0.20493 (12)	0.4373 (5)	0.7670 (3)	0.0461 (6)	

### Atomic displacement parameters (Å^2^)

**Table:**

	*U*^11^	*U*^22^	*U*^33^	*U*^12^	*U*^13^	*U*^23^
N1	0.0276 (8)	0.0464 (12)	0.0496 (12)	0.0045 (7)	0.0001 (8)	−0.0074 (9)
N2	0.0280 (9)	0.0437 (10)	0.0402 (10)	−0.0004 (7)	−0.0032 (8)	−0.0026 (9)
N3	0.0318 (10)	0.1012 (17)	0.0592 (14)	0.0168 (9)	0.0022 (10)	0.0077 (13)
C1	0.0264 (9)	0.0338 (9)	0.0378 (12)	−0.0003 (7)	0.0017 (8)	0.0061 (9)
C2	0.0266 (8)	0.0356 (10)	0.0329 (11)	−0.0004 (7)	−0.0006 (8)	0.0070 (8)
C3	0.0300 (9)	0.0424 (11)	0.0338 (11)	0.0046 (8)	0.0028 (9)	0.0049 (10)
C4	0.0265 (9)	0.0503 (13)	0.0375 (12)	0.0044 (8)	0.0010 (9)	0.0125 (10)
C5	0.0290 (10)	0.0517 (12)	0.0418 (13)	−0.0043 (8)	−0.0038 (10)	0.0077 (11)
C6	0.0354 (11)	0.0431 (11)	0.0408 (12)	−0.0012 (8)	0.0004 (10)	−0.0021 (10)
C7	0.0323 (11)	0.0666 (14)	0.0393 (13)	0.0045 (9)	0.0013 (10)	0.0087 (11)

### Geometric parameters (Å, º)

**Table:**

N1—C1	1.378 (3)	C2—C3	1.382 (3)
N1—H1A	0.91 (3)	C3—C4	1.399 (3)
N1—H1B	0.92 (3)	C3—H3	0.9500
N2—C2	1.406 (3)	C4—C5	1.388 (3)
N2—H2A	0.95 (3)	C4—C7	1.433 (3)
N2—H2B	0.89 (3)	C5—C6	1.383 (3)
N3—C7	1.148 (3)	C5—H5	0.9500
C1—C6	1.399 (3)	C6—H6	0.9500
C1—C2	1.416 (3)		
			
C1—N1—H1A	120.5 (19)	C2—C3—H3	119.5
C1—N1—H1B	113.8 (16)	C4—C3—H3	119.5
H1A—N1—H1B	120 (2)	C5—C4—C3	119.93 (16)
C2—N2—H2A	112.7 (19)	C5—C4—C7	119.96 (18)
C2—N2—H2B	111.3 (17)	C3—C4—C7	120.1 (2)
H2A—N2—H2B	115 (3)	C6—C5—C4	119.51 (19)
N1—C1—C6	120.0 (2)	C6—C5—H5	120.2
N1—C1—C2	120.94 (17)	C4—C5—H5	120.2
C6—C1—C2	119.04 (17)	C5—C6—C1	121.3 (2)
C3—C2—N2	121.9 (2)	C5—C6—H6	119.3
C3—C2—C1	119.10 (17)	C1—C6—H6	119.3
N2—C2—C1	118.95 (17)	N3—C7—C4	178.4 (3)
C2—C3—C4	121.1 (2)		

### Hydrogen-bond geometry (Å, º)

**Table:**

*D*—H···*A*	*D*—H	H···*A*	*D*···*A*	*D*—H···*A*
N1—H1*A*···N3^i^	0.91 (3)	2.49 (3)	3.218 (3)	138 (2)
N1—H1*B*···N2^ii^	0.92 (3)	2.20 (3)	3.107 (3)	170 (3)
N2—H2*A*···N3^i^	0.95 (3)	2.22 (3)	3.152 (3)	168 (3)
N2—H2*B*···N1^iii^	0.89 (3)	2.41 (3)	3.210 (3)	149 (2)

Symmetry codes: (i) *x*+1/2, −*y*+1, *z*; (ii) −*x*+1, −*y*, *z*−1/2; (iii) −*x*+1, −*y*+1, *z*+1/2.
